# Five-Year Prospective Observational Study of African-American Men on Active Surveillance for Prostate Cancer Demonstrates Race Is Not Predictive of Oncologic Outcomes

**DOI:** 10.1093/oncolo/oyac154

**Published:** 2022-08-03

**Authors:** Joshua Pincus, Jacob W Greenberg, Caleb Natale, Christopher R Koller, Stephanie Miller, Jonathan L Silberstein, L Spencer Krane

**Affiliations:** Department of Urology, Tulane University School of Medicine, New Orleans, LA, USA; Department of Urology, Tulane University School of Medicine, New Orleans, LA, USA; Department of Urology, Tulane University School of Medicine, New Orleans, LA, USA; Department of Urology, Tulane University School of Medicine, New Orleans, LA, USA; Southeast Louisiana Veterans Health Care System, New Orleans, LA, USA; Memorial Healthcare System, Aventura, FL, USA; Department of Urology, Tulane University School of Medicine, New Orleans, LA, USA; Southeast Louisiana Veterans Health Care System, New Orleans, LA, USA

**Keywords:** prostate cancer, active surveillance, health care disparities, African American, progression-free survival, outcomes

## Abstract

**Introduction:**

This study aimed to evaluate if race impacted outcomes or risk of disease progression in men on active surveillance (AS) for prostate cancer. We present the results from our majority African-American cohort of men in an equal access setting over a 5-year follow-up period.

**Patients and Methods:**

All patients who elected AS for prostate cancer at the Southeast Louisiana Veterans Health Care System are entered into a prospectively managed observational database. Patients were divided into groups based on self-reported race. Grade group progression was defined as pathologic upgrading above International Society of Urological Pathology Grade Group 1 disease on subsequent biopsies following diagnostic biopsy. All tests were 2 sided using a significance of .05.

**Results:**

A total of 228 men met inclusion criteria in the study, including 154 non-Hispanic African American and 74 non-Hispanic Caucasian American men, with a median follow-up of 5 years from the initiation of AS. Race was not predictive of Gleason grade progression, AS discontinuation, or biochemical recurrence on Cox multivariate analysis (HR = 1.01, 0.94, 0.85, *P* = .96, .79, .81, respectively). On Kaplan-Meier analysis at 5 years, African-American progression-free, AS discontinuation free, and overall survival probability was comparable to their Caucasian American counterparts (*P* > .05 for all).

**Conclusions:**

Active surveillance is a safe treatment option for low and very low risk prostate cancer, regardless of race. African-American and Caucasian-American men did not have any significant difference in Gleason grade group progression in our cohort with 5-year follow-up.

## Teaser

Men of African American heritage have been historically excluded from active surveillance (AS), considered the most conservative treatment option for those diagnosed with prostate cancer. This article evaluates whether race is predictive of AS in an equal access to care setting, with a 5-year follow-up. Race was not found to be a predictor and AS can safely be offered to AA men diagnosed with low risk prostrate cancer.

**Implications for Practice**

Historically, men of African-American heritage (AA) with low-risk prostate cancer have been excluded from active surveillance (AS) as a viable treatment option due to a perceived increased risk of harboring highly neoplastic disease. However, this assumption is based on research with few enrolled AA patients, largely retrospective in nature, and data confounded by socioeconomic stressors. In this study conducted at a single-payer health care system, race was not associated with AS failure, worse treatment outcomes, or biochemical recurrence. This study highlights that AA men with low-risk disease can safely be offered AS due to comparable outcomes with non-AA counterparts.

## Introduction

Prostate cancer is the most common non-skin malignancy diagnosed and second leading cause of cancer-related death in men within the US.^1^ The incidence to mortality ratio for prostate cancer remains higher than any other malignancy, and men with low-risk prostate cancer have a greater risk of dying from causes other than prostate cancer, even 20 years after diagnosis.^2^ Curative local therapies for prostate cancer have established side effect profiles including urinary incontinence and erectile dysfunction. Active surveillance (AS) involves monitoring of localized prostate cancer with the intent of preventing unnecessary treatment while still addressing clinically significant disease during the window of curability.^3-5^ Long-term data regarding the safety of AS continue to produce excellent prostate cancer-specific outcomes.^6^ African-American (AA) race is a known risk factor for prostate cancer diagnosis, advanced stage prostate cancer at time of diagnosis, and prostate cancer-specific mortality.^7^ Concerns regarding these increased risks have prevented universal acceptance of AS in AA populations with prostate cancer.^8^ Providing extended follow up outcomes is vital to encouraging AS for AA men with low risk disease.^9,10^

At the Southeast Louisiana Veterans Health Care System (SLVHCS), AA men have equal access to prostate cancer care as the non-AA members of this community. Here we present the results from our AS cohort over a 5-year follow-up period. We aim to evaluate if race impacts prostate cancer outcomes or risk of disease progression in men electing AS in an equal access to care setting.

## Patients and Methods

We maintain an Institutional Review Board (IRB) approved dataset of all men electing to undergo prostate biopsies at the SLVHCS in New Orleans, Louisiana beginning in 2010 (IRB #563-629). At our institution, we encourage all patients with NCCN very low risk category or low risk category prostate cancer and expected survival of at least 10 years to pursue AS. We included all males aged 18 and older receiving a biopsy at SLVHCS between January 1, 2010, and October 5, 2021, enrolled on AS as their first-line treatment following a diagnostic biopsy demonstrating International Society of Urological Pathology Grade Group 1 (GG1) prostate cancer, having received a confirmatory biopsy within 12 months of initial diagnosis, and ≥10-year life expectancy. Race was self-reported to the Veterans Health Administration (VHA). Positive family history was defined as the subject having a first-degree male relative previously diagnosed with prostate cancer.

### Patient Management

**Figure 1.**
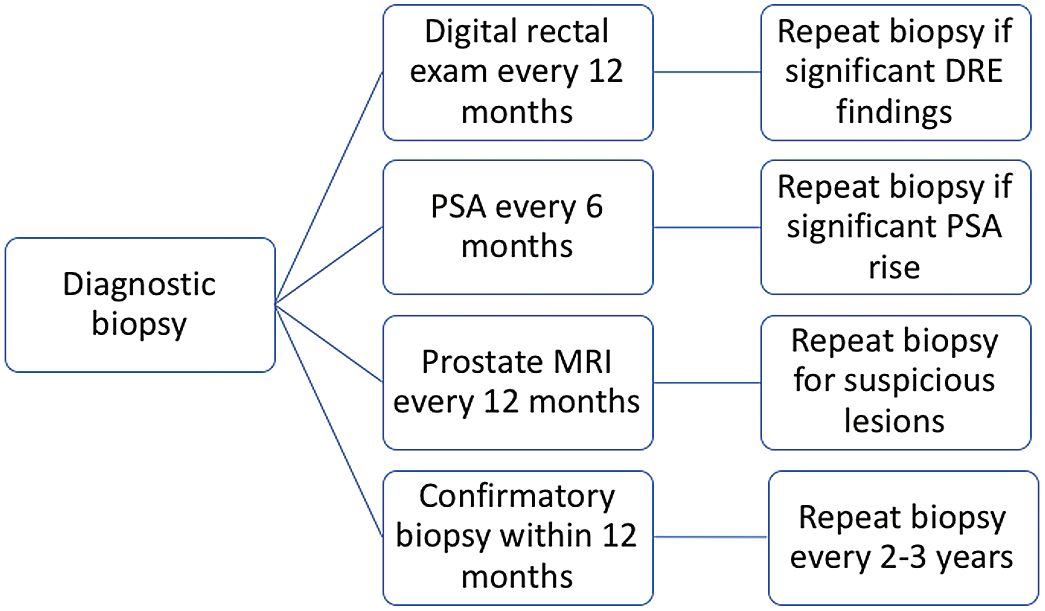
Active surveillance protocols at the SLVHCS—PSA, MRI, and DRE are performed on a scheduled basis, and biopsies are repeated every 2-3 years or for cause.

The AS protocol at the SLVHCS includes prostate-specific antigen (PSA) testing and digital rectal exam (DRE) performed at each 6-month interval (Fig. 1). A confirmatory biopsy is performed within 12 months of the diagnostic biopsy and repeated every 2-3 years or for cause. For cause biopsy was defined as rapid change in PSA, change in DRE, or new radiographic findings. All biopsies included at least 12 cores with ultrasound guidance. MRI fusion biopsies began in 2017 at our institution and were then uniformly performed in this cohort. Grade Group progression was defined as pathologic upgrading above GG1 disease on confirmatory or repeat biopsy. Men with grade group progression were recommended to undergo definitive treatment for prostate cancer. Biochemical recurrence (BCR) was defined as PSA 2.0 plus the nadir for patients undergoing radiation therapy and 2 PSA values >0.05 for those choosing radical prostatectomy (RP). All pathology was reviewed by fellowship trained pathologists at SLVHCS. If concerns existed regarding pathologic diagnosis, the samples were sent for central pathologic review at the Joint Pathology Center.

### Statistical Methods

Upon data lock on October 5, 2021, pathologic, clinical, and laboratory data were correlated with outcomes variables. Non-parametric data was reported in medians, while means were used if variables were parametrically distributed. Using R version 4.1.2 (Ann Arbor, MI), Kaplan-Meier (KM) plots including grade group progression-free and AS discontinuation-free probability were created. Hazard ratios (HR) were calculated through a multivariable Cox proportional hazards regression analyses. KM curves and HR values were generated using R package “survivalAnalysis” and “survminer.” Stacked bar graphs were made using R package “ggplot2” and “tidyverse.” Fisher’s exact and chi-square tests were used to compare categorical data between races. Due to the non-parametric nature of this study’s data, Mann-Whitney U tests were used to compare various continuous variables between racial groups. All tests were 2-sided when applicable with a significance level of 0.05. A CI of 95% was used when reporting survival probability and HR.

### Outcomes

The primary outcome of this study was time to grade group progression or AS discontinuation. Two secondary outcomes were identified: the rate of grade group progression on AS between AA and CA patients and treatment outcomes and BCR between races. Time on AS was calculated for timespan between date of first prostate cancer diagnosis to last follow-up or PSA testing given no grade group progression has occurred. Time to grade group progression was calculated as the timespan between date of first prostate cancer diagnosis to date of grade group progression on biopsy pathology. Time to AS discontinuation was calculated as the timespan between date of first prostate cancer diagnosis to date of grade group progression or elective treatment.

## Results

### Patient Demographics and Gleason Grade Progression Rates

From a database of 310 veterans on AS, 228 patients met our inclusion criteria. 82 were excluded due to NCCN risk of intermediate through high and/or absence of confirmatory biopsy. Included subjects were grouped by self-reported race, with 154 non-Hispanic African American and 74 non-Hispanic Caucasian men (Table 1). Protocol compliance rate for the whole cohort was found to be 60.1%. Furthermore, racial groups, AA and CA men, were found to be equally compliant to the AS protocol. At the time of diagnosis, AA and CA men were found to be comparable in respect to age, body mass index (BMI), smoking status, number of cores taken, family history, PSA, prostate volume, PSA density (PSAD), number of positive cores, clinical T stage, and NCCN risk category (all *P* > .05). Equivalent results were identified on confirmatory biopsy. Over the course of this study a total of 107 patients experienced grade group progression, 77 (50%) AA and 30 (41%) CA. A Fisher’s exact test comparing overall grade group progression rates between races yielded a *P*-value of .20. AA and CA men were followed for a median of 5.3 and 5.0 years, respectively (*P* = .16).

**Table 1.** Demographic statistics.

	Diagnostic biopsy			Confirmatory biopsy		
	AA	CA	*P*-value	AA	CA	*P*-value
Number (%)	154 (68%)	74 (32%)	–	154 (68%)	74 (32%)	–
Median PSA ng/mL (IQR)	5.43 (4.64-6.76)	5.11 (4.33-6.44)	.15	5.66 (4.14-7.33)	5.05 (4.03-6.93)	.12
Median PSA density (IQR)	0.15 (0.09-0.2)	0.13 (0.088 – 0.21)	.39	0.17 (0.09-0.24)	0.13 (0.09-0.21)	.23
Median prostate volume (IQR)	39.3 (29.9-55)	42.1 (28.6-56.7)	.56	38.3 (27.7-53.6)	38.5 (32.1-56.4)	.26
Median number positive cores (IQR)	2 (1-3)	1.5 (1 – 2)	.71	1 (0 – 3)	1 (0 – 3)	.56
Number positive cores (%)	–	–	–	–	–	–
0	0 (0%)	0 (0%)	–	47 (31%)	25 (34%)	–
1	76 (50%)	38 (51%)	–	35 (22%)	14 (19%)	–
2	36 (23%)	19 (26%)	–	21 (14%)	12 (16%)	–
3	22 (14%)	9 (12%)	–	15 (10%)	8 (11%)	–
>3	20 (13%)	8 (11%)	.92	36 (23%)	15 (20%)	.90
Gleason score (%)	–	–	–	–	–	–
No cancer on repeat	0 (0%)	0 (0%)	–	47 (31%)	25 (34%)	–
GG1	154 (100%)	74 (100%)	–	62 (40%)	28 (38%)	–
GG2	0 (0%)	0 (0%)	–	23 (15%)	12 (16%)	–
GG3 or greater	0 (0%)	0 (0%)	–	22 (14%)	9 (12%)	.93
Median age at diagnosis (IQR)	65 (61-68)	65 (61-68.8)	.95	–	–	–
Baseline no. T stage (%)	–	–	–	–	–	–
T1c	139 (90%)	62 (84%)	–	–	–	–
T2a	15 (10%)	12 (16%)	.19	–	–	–
Median BMI (IQR)	28.5 (25-32)	28.3 (25.4-32.6)	.65	–	–	–
Family history of PCa	34 (22%)	11 (15%)	.22	–	–	–
Smoker status	–	–	–	–	–	–
Non	70 (45%)	31 (42%)	–	–	–	–
Current/former	84 (55%)	43 (58%)	.67	–	–	–
NCCN guideline category	–	–	–	–	–	–
Very low	53 (34%)	31 (42%)	–	–	–	–
Low	101 (66%)	43 (58%)	.30	–	–	–
Overall length from AS to follow up	5.3 years	5.0 years	.16	–	–	–
Gleason upgraded by NCCN at Dx Bx	–	–	–	–	–	–
Very low	17 (32%)	8 (26%)	.63	–	–	–
Low	60 (59%)	22 (52%)	.37	–	–	–

There was no significant difference between groups with respect to demographic data or prostate cancer characteristics at diagnosis or on confirmatory biopsy.

Abbreviations: GG, grade group; AA, African American; CA, Caucasian.

All patients included in this study underwent a confirmatory biopsy. Furthermore, racial groups were found to have undergone comparable numbers of overall biopsies. 91 (43% AA, 29% CA, *P* = .12) underwent a third overall biopsy, 35 (34% AA, 29% CA, *P* = .80) had 4, and 7 (3%) undergoing ≥5 overall biopsies following initiation of AS. Racial groups underwent comparable numbers of overall biopsies. On confirmatory biopsy, 32% (31% AA, 34% CA) of patients had a benign biopsy; 40% (40% AA, 38% CA) had stable GG1 disease, 15% (15% AA, 16% CA) had upgraded GG2 disease, and 14% (14% AA, 12% CA) had upgraded ≥GG3 disease. A contingency table between racial groups and Gleason grade on confirmatory biopsy yielded a *P*-value of .93. A Fisher’s exact test for grade group progression at third overall biopsy between our racial groups yielded respective *P*-values of .99.

Forty percentage (43% AA, 29% CA, *P* = .12) of patients further went on to receive 4 overall biopsies. Forty-one percentage (43% AA, 32% CA) were found to have benign histology, 26% (28% AA, 21% CA) had stable GG1, 19% (21% AA, 13% CA) had upgraded GG2, and 14% (13% AA, 17% CA) had upgraded ≥GG3 disease. On histologic evaluation, AA and CA men were found to have comparable Gleason grade disruption. Additionally, a Fisher’s exact test for grade group progression between racial groups (34% AA, 29% CA) yielded a *P*-value of .80. Thirty-five men went on to receive 5 overall biopsies with comparable rates of grade group progression between races on Fisher’s exact test (*P* = .46).

### Kaplan-Meier Analyses for Racial Variation in Grade Group Progression-Free Survival

**Figure 2.**
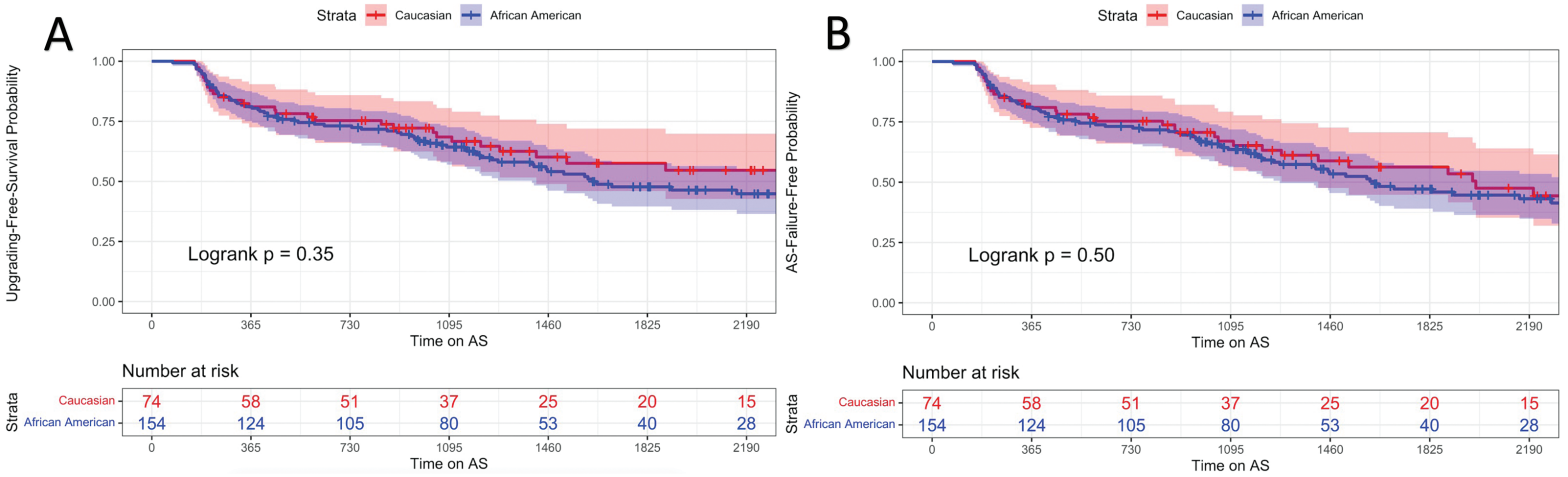
(**A**) Kaplan-Meier curve of grade group grogression-free survival probability by race—there is no significant difference between AA and CA groups with respect to survival of grade group progression on AS. (**B**) Kalpan-Meier curve for discontinuing-AS-free probability by race—AA and CA men had comparable AS protocol survival.

KM curves were generated to analyze the difference in grade group progression-free and AS discontinuation-free survival between races. At 2.5 years, the grade group progression-free survival probability was 71% (CI 63%-78%) for AA men and 72% (CI 63%-83%) for CA men (Fig. 2A). At 5 years, the grade group progression-free survival probability was 48% (CI 40-58%) for AA men and 58% (CI 4-6-72%) for CA men. No difference between CA and AA men grade group progression-Free survival was identified (log-rank *P* = .35). At 2.5 years, the AS discontinuation-free survival probability was 68% (CI: 60%-75%) for AA men and 69% (CI: 59%-81%) for CA men (Fig. 2B). At 5 years, the AS discontinuation-free survival 44% (CI: 36%-54%) for AA men and 50% (CI 68%-39%) for CA men. No differences between CA and AA men AS discontinuation-free survival were identified (log-rank *P* = .50).

**Figure 3.**
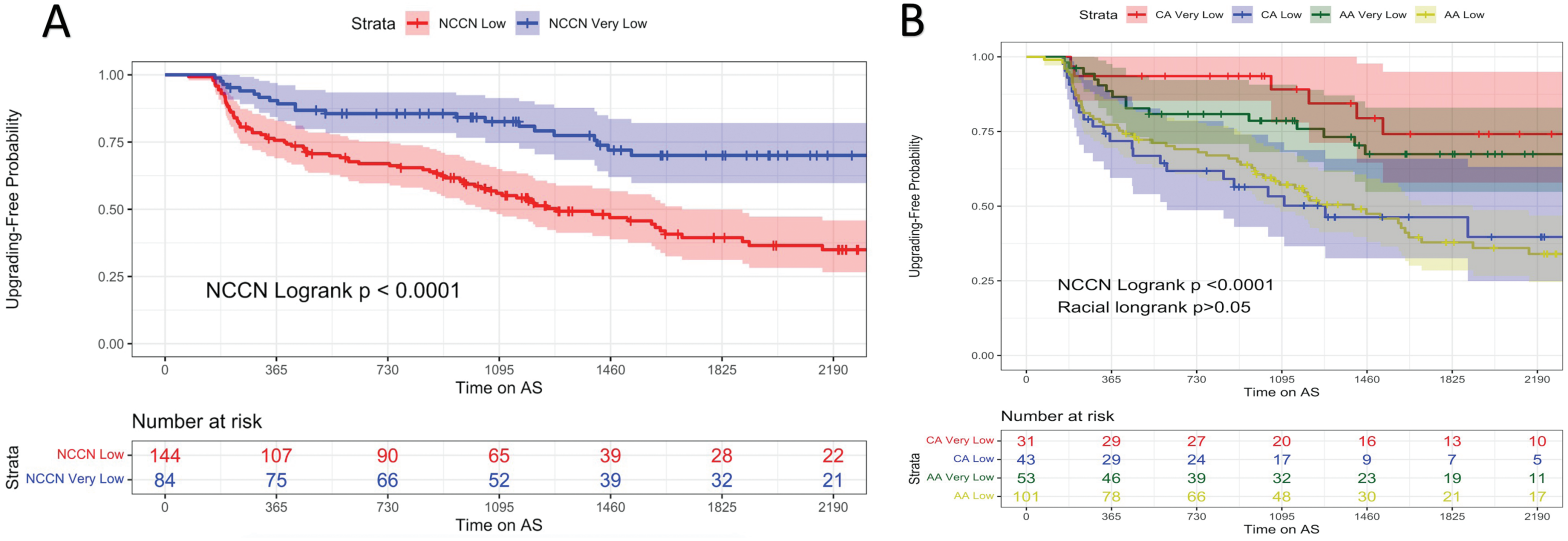
(**A**) Kaplan-Meier curve of grade group progression-free survival probability by NCCN risk classification—there is a significant difference between risk classification groups. Patients with NCCN very low risk disease displayed lower grade group progression-free survival than subjects diagnosed with NCCN low-risk disease. (**B**) Kaplan-Meier curve of grade group progression-free survival probability by race and NCCN risk classification—there is no difference between race groups adjusted for risk classification.

We validated these findings in grade group progression-free survival by splitting our cohort by NCCN risk category (Fig. 3A). The NCCN low risk cohort had a significantly lower grade group progression-free survival compared to very-low risk patients (log-rank *P* < .0001). We further elucidated any racial differences in grade group progression-free survival by delineating the NCCN risk categories by race (Fig. 3B). There was no significant difference in grade group progression-free survival between races within each NCCN risk classification (all *P* > .05).

### Cox Multivariable Analysis for Gleason Grade Progression, Discontinuation of AS, and Biochemical Recurrence

Patient survival data were used to generate Cox multivariable proportional hazards models (MVA). This study used 3 distinct outcome variables: grade group progression, AS discontinuation, and BCR (Table 2). The whole patient cohort was included when outcome variables grade group progression and AS discontinuation were used. Only patients who underwent definitive treatment were included in the MVA for BCR. Race was not found to be a predictive grade group progression, AS discontinuation, or BCR (HR = 1.01, 0.94, 0.85, *P* = .96, 0.79, 0.81, respectively). Age, BMI, history of a benign prostate biopsy prior to diagnosis, family history of prostate cancer, and PSA were also not predictive across all 3 MVAs (All *P* > .05). Number of positive cores at diagnostic biopsy was positively predictive of both grade group progression and AS discontinuation (*P* < .0001), but not BCR (*P* = .57). Very low risk disease was protective against grade group progression (HR = 0.58 [CI: 0.3-4-0.99], *P* = .047) and AS discontinuation (HR = 0.60 [CI: 0.3-6-0.98), *P* = .041). PSAD was also positive predictive of AS discontinuation.

**Table 2.** Cox multivariable regression analysis*—*multivariable analysis for Gleason progression and discontinuing AS was done on the whole cohort of patients (*n* = 228).

	Gleason progression		Discontinue AS		Biochemical recurrence	
Variable	Hazard ratio (95% CI)	*P*-value	Hazard ratio (95% CI)	*P*-value	Hazard ratio (95% CI)	*P*-value
Age at Dx	1.02 (0.99-1.05)	.21	1.01 (0.98-1.04)	.58	0.99 (0.88-1.09)	.70
BMI	1.01 (0.9-7-1.04)	.63	1.02 (0.98-1.05)	.34	0.93 (0.82-1.05)	.25
Race	–	–	–	–	–	–
AA	1.01 (0.64-1.59)	.96	0.94 (0.62-1.43)	.76	0.85 (0.22-3.33)	.81
CA	Referent	–	Referent	–	Referent	–
PSA at Dx	0.97 (0.85-1.10)	.60	0.98 (0.86-1.10)	.68	1.10 (0.71-1.71)	.67
PSAD at Dx	3.01 (0.73-12.4)	.13	3.84 (1.05-13.9)	.042	–	–
No. of positive cores at Dx	1.33 (1.19-1.50)	<.0001	1.32 (1.19-1.47)	<.0001	1.10 (0.80-1.50)	.57
NCCN risk	–	–	–	–	–	–
Very low	0.58 (0.34-0.99)	.047	0.60 (0.36-0.98)	.041	2.92 (0.74-11.5)	.13
Low	Referent	–	Referent	–	Referent	–
Family Hx of PCa	0.95 (0.58-1.56)	.84	1.05 (0.66-1.65)	.85	0.82 (0.20-3.37)	.78
Benign Bx before Dx	1.23 (0.75-2.03)	.41	1.02 (0.63-1.68)	.92	0.32 (0.04-2.68)	.29

Multivariable analysis for biochemical recurrence was conducted on patients who underwent definitive treatment after discontinuing AS. There was no significant difference between AA and CA groups with respect to Gleason progression, AS discontinuation, or biochemical recurrence.

Abbreviations: Dx, diagnosis; BMI, body mass index; AA, African American; CA, Caucasian; PSA, prostate specific antigen; PSAD, prostate specific antigen density; NCCN, National Comprehensive Cancer Network; Bx, biopsy.

### Treatment Outcomes and Rate of Biochemical Recurrence

One hundred and nine out of the 228 patients included underwent definitive treatment (Table 3). AA and CA patients underwent RP and medical/focal treatment at comparable rates (*P* = .28). Furthermore, when analyzing histologic features after RP, Gleason grade group distribution, pT staging, pN staging, and pM staging were statistically similar between racial groups. 12 patients experienced BCR, all of whom received salvage therapy.

**Table 3.** Treatment and outcomes.

Treatment outcomes, *n* (%)	AA	CA	*P*-value
Tx for grade progression	72 (47%)	25 (34%)	–
Elective treatment	7 (5%)	5 (7%)	.31
Radical prostatectomy	42 (53%)	12 (40%)	–
Medical or focal intervention	37 (47%)	18 (60%)	.28
External beam radiation	30 (81%)	15 (83%)	–
Brachytherapy	3 (8%)	1 (6%)	–
Ablation	3 (8%)	2 (11%)	–
ADT alone	1 (3%)	0 (0%)	–
Unknown pathology results	2 (5%)	0 (0%)	.99
pT stage	–	–	–
T2	25 (63%)	9 (75%)	–
T3	15 (37%)	3 (25%)	.51
pN stage	–	–	–
N0	40 (100%)	12 (100%)	–
N1	0 (0%)	0 (0%)	1.00
pM stage	–	–	–
M0	40 (100%)	12 (100%)	–
M1	0 (0%)	0 (0%)	1.00
RP pathology GG	–	–	–
1	4 (10%)	2 (17%)	–
2	24 (60%)	9 (75%)	–
3	10 (25%)	1 (8%)	–
5	2 (5%)	0 (0%)	.74
Biochemical recurrence	9 (13%)	3 (12%)	.99

A comparable number of AA and CA patients underwent treatment for Gleason progression or elective reasons. Additionally, when comparing medical/focal vs radical proctectomy, pT stage, pN stage, pM stage, Gleason grade group on RP, and biochemical recurrence, racial groups showed no difference.

Abbreviations: AA, African American; CA, Caucasian; Tx, Treatment; pT, pathologic T stage; pN, pathologic N stage; pM, pathologic M stage; RP, radical prostatectomy; GG, grade group; ADT, androgen deprivation therapy.

### Metastasis Free, Prostate Cancer, and Overall Survival

Over the course of study, 26 (11%) patients have passed from causes unrelated to prostate cancer, with 13 passing since the start of the COVID-19 pandemic. Nine of these patients were CA and 17 AA with a Fisher’s exact *P*-value of .83. Zero patients have died to causes relating to their Prostate cancer. None of the patients who passed had spread of prostate cancer to either lymph node or distant metastases. Two hundred and two patients were alive at the time of the data-lock, and 5-year survival probability (Supplementary Fig. S1) was 94% (CI 90%-97%) with no difference in race (*P* = .69).

## Discussion

Current data on the safety of AS for AA men are limited. Several studies have shown increased risk of grade group progression and AS discontinuation for AA men on AS, including a recent retrospective study by Deka et al and prospective cohort from Tosoian et al that followed >2000 and 151 AA men, respectively.^11-15^ In contrast, there are also multiple cohort studies that have demonstrated no difference in grade group progression and AS discontinuation between AA and CA men, such as the prospective MD Anderson study by Davis, et al, and the prospective Canary PASS study.^9,16-19^ Unfortunately, these studies share the limitations of short follow-up and small cohort size of AA men. A recent meta-analysis by Vigneswaran et al including 12 such studies, totaled 3137 AA and 12,206 non-AA men. These researchers found that AA men with early-stage and grade prostate cancer have a higher risk for grade group progression than non-AA men.^7^ However, they also noted that the increased risk has decreased over time. While there is an established debate over AA risk on AS, few published studies have considered the effect of access to health care on their results. Within the VHA, barriers to health care access are minimized. Multiple studies in equal access settings such as military or VHA settings did not find race-specific differences in grade group progression or prostate cancer survival.^7,9,20^

This study adds to the current literature by evaluating racial disparities in the setting of an equal access facility. With over 5 years of follow-up, AA race was not predictive of AS discontinuation, grade group progression, or BCR. On KM analysis, AA displayed comparable grade group progression free, AS discontinuation free, prostate cancer specific, and overall survival at 2.5 and 5 years, even when delineating by NCCN risk category. Finally, after analyzing RP Gleason grade and pathologic staging, AA and CA men continued to display comparable outcomes.

Our study has several important strengths. This is the largest published single institutional series of AA men who have elected AS and been followed for an extended duration. Most single institution series continue to have limited AA patients. In this majority AA cohort, our data supports safety of AS for these patients, and assists clinicians with counseling of these patients who are less likely to pursue AS.^21^ Our protocol used strict, clear inclusion criteria, follow-up regiments, and used objective pathologic criteria to define outcomes. This cohort is more representative of a “real world” example of patients electing AS. Our overall rate of AS discontinuation (53%) is consistent with a recent observational population-based study published in *Journal of Urology* (51%).^22^

The primary limitation of our study is that this is a single institution analysis. Despite this, we believe this to be more representative of the AS cohorts seen by practicing urologists. The published literature tracks encounters from major academic centers with well-resourced patients. While not assessed directly in this study, a previous analysis from the southern US revealed that patients with prostate cancer demonstrate poor health literacy, which is associated with more mental distress.^23^ Furthermore, Coughlin et al highlighted poor literacy nationwide, and in particular many AA men have inadequate knowledge of prostate cancer.^24^ We believe that when counseling patients with low health literacy and low risk disease, providing robust data to support AS is key to encouraging less aggressive local disease control. Another limitation is alterations in our AS protocols due to technological advancement. Initially we relied upon template biopsies for surveillance, but we now have incorporated multiparametric MRI and MRI fusion biopsies.^25^ AA and non-AA men were shown to have comparable rates of multiparametric MRI utilization in this study and this follows national trends in increasing MRI utilization for AS protocols. It must also be noted that while this data represents excellent 5-year oncologic outcomes, long-term follow-up is needed. It is the prevailing opinion of the authors that because an equal access care cohort showed short (2.5 years) and intermediate (5 years) term comparable racial outcomes, long-term data are hypothesized to further validate this investigation.

Nationwide, AA men are still enrolled on AS at lower rates than CA men.^26^ Physicians have veered away offering AA men AS due to prior literature suggesting an increased risk of metastatic disease, and mortality.^27-29^ However, this study bolsters the growing evidence that AS remains a safe and effective management strategy for African-American patients with localized very low and low-risk prostate cancer. When socioeconomic pressures to health care are minimized, race does not contribute to AS oncologic outcomes. There is much we can improve upon as a society in our treatment of AA men with prostate cancer, including improving access to affordable health care, equal representation in clinical studies, and offering AA men equal access to all treatment options, including AS, which this study has demonstrated to be a safe option for very low and low-risk prostate cancer regardless of race.

## Conclusion

African-American men with NCCN very low and low-risk prostate cancer can safely elect an AS protocol with comparable outcomes to their Caucasian counterparts. When compared with non-African American men, African-American men have no significant increased risk of grade group progression, worse pathological staging after treatment, or BCR. Providers should not discourage men from choosing AS for NCCN very low and low-risk prostate cancer based solely on race.

Current disparities in prostate cancer care are and should be a major concern for all providers including urologists, genitourinary and general oncologists, and primary care physicians, among many. Time and time again, access to care included in social determents of health has been shown to be a powerful predictor of health care outcomes with some publications reporting a representation of up to 90%.^30,31^ Historically, prior investigations have shown that men of African-American heritage fail AS protocols at higher rates and harbor higher risk disease than their non-AA counterparts. However, these publications are confounded by the socioeconomic pressures of the US health care system and do not account for access to care. This investigation’s findings display that when access to care is no longer a contributing risk factor, men of African-American heritage have comparable oncologic outcomes to non-AA patients. When interacting with patients of diverse backgrounds, oncology care providers should reflect on their own implicit biases and provide evidence-based care to all patients.

## Supplementary Material

oyac154_suppl_Supplementary_Figure

## Data Availability

The data underlying this article will be shared on reasonable request to the corresponding author.
