# Supplementary figures and images for: Five-Year Prospective Observational Study of African-American Men on Active Surveillance for Prostate Cancer Demonstrates Race Is Not Predictive of Oncologic Outcomes
Source: Oncologist. 2022 Aug 3;28(2):149–56. doi: 10.1093/oncolo/oyac154 (PMC9907040; doi:10.1093/oncolo/oyac154)

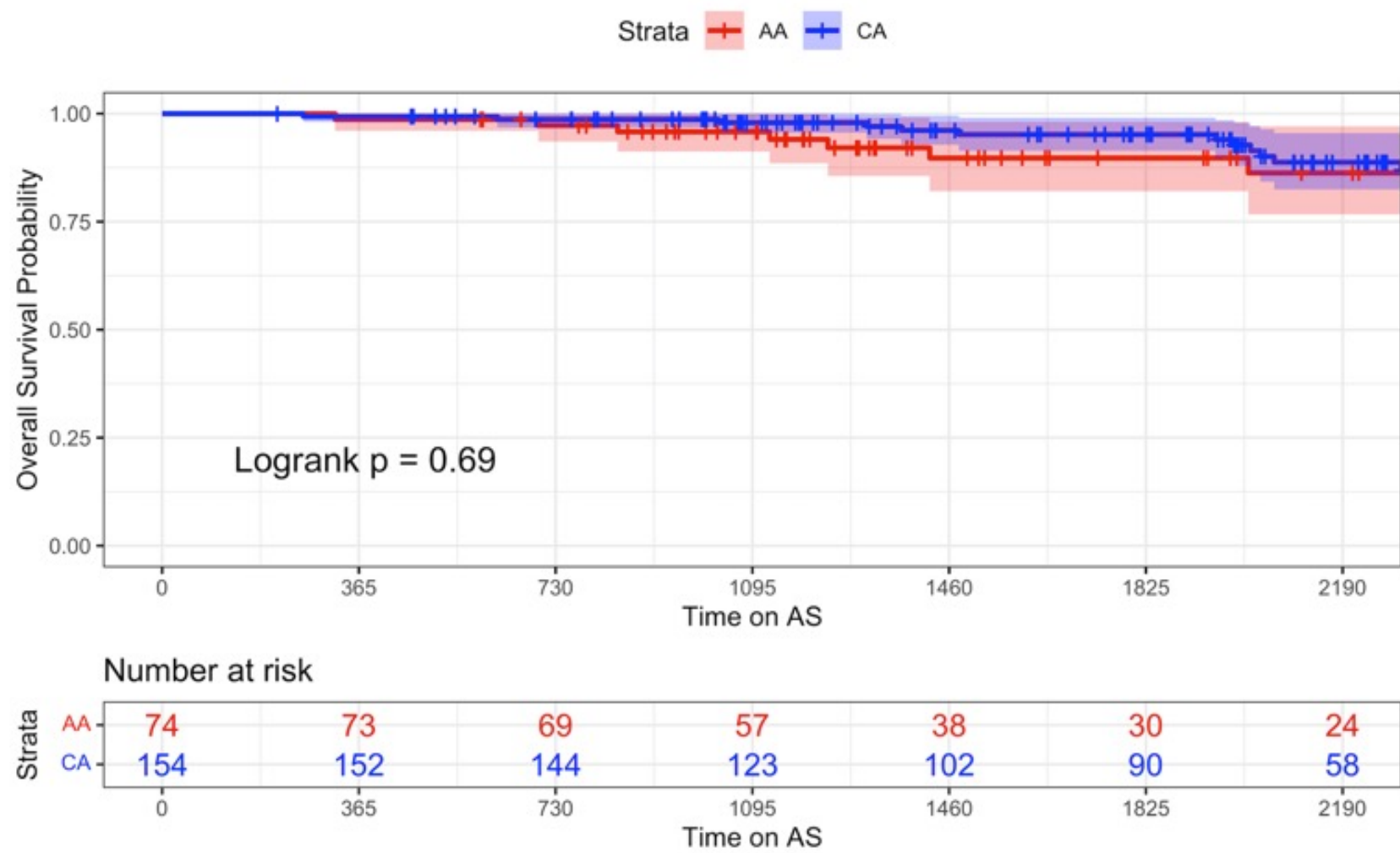

Supplement: oyac154_suppl_Supplementary_Figure [file oyac154_suppl_supplementary_figure.pdf]
